# An Intra-COVID-19 Assessment of Hand Hygiene Facility, Policy and Staff Compliance in Two Hospitals in Sierra Leone: Is There a Difference between Regional and Capital City Hospitals?

**DOI:** 10.3390/tropicalmed6040204

**Published:** 2021-11-29

**Authors:** Sulaiman Lakoh, Emmanuel Firima, Christine Ellen Elleanor Williams, Sarah K. Conteh, Mohamed Boie Jalloh, Mohamed Gbeshay Sheku, Olukemi Adekanmbi, Stephen Sevalie, Sylvia Adama Kamara, Mohamed Akmed Salim Kamara, Umu Barrie, Gladys Nanilla Kamara, Le Yi, Xuejun Guo, Chukwuemeka Haffner, Matilda N. Kamara, Darlinda F. Jiba, Enanga Sonia Namanaga, Anna Maruta, Christiana Kallon, Joseph Sam Kanu, Gibrilla F. Deen, Mohamed Samai, Joseph Chukwudi Okeibunor, James B. W. Russell

**Affiliations:** 1 College of Medicine and Allied Health Sciences, University of Sierra Leone, Freetown, Sierra Leone; mboie1537@gmail.com (M.B.J.); stevesyllo@gmail.com (S.S.); kamaramatilda9198@gmail.com (M.N.K.); samjokanu@yahoo.com (J.S.K.); gibrilladeen1960@yahoo.com (G.F.D.); dhmsamai@yahoo.com (M.S.); jamesbwrussell@gmail.com (J.B.W.R.); 2 Ministry of Health and Sanitation, Government of Sierra Leone, Freetown, Sierra Leone; ceewilliams54@yahoo.com (C.E.E.W.); contehsk@gmail.com (S.K.C.); mgsheku42@gmail.com (M.G.S.); haffnerbarnaby@gmail.com (C.H.); darlindajiba.dj@gmail.com (D.F.J.); enangasonia@yahoo.com (E.S.N.); christy.conteh@yahoo.com (C.K.); 3 Clinical Research Unit, Department of Medicine, Swiss Tropical and Public Health Institute, CH-4051 Basel, Switzerland; 4 University of Basel, CH-4001 Basel, Switzerland; 5 SolidarMed, Christie House 3rd Floor, Orpen Road, Old Europa, P.O. Box 0254, Maseru West 105, Lesotho; 6 34 Military Hospital, Freetown, Sierra Leone; adama092286@gmail.com (S.A.K.); mohamedakmedsalimkamara@yahoo.com (M.A.S.K.); gladysnanillak14@gmail.com (G.N.K.); 7 Department of Medicine, College of Medicine, University of Ibadan, Ibadan 200005, Nigeria; kemiosinusi@gmail.com; 8 Department of Medicine, University College Hospital, Ibadan 200005, Nigeria; 9 Infectious Disease Research Network, Freetown, Sierra Leone; barrieumu1993@gmail.com; 10 Changchun Veterinary Research Institute, Chinese Academy of Agricultural Sciences, Changchun 130122, China; lohryee@gmail.com (L.Y.); xuejung@yahoo.com (X.G.); 11 World Health Organization Country Office, Freetown, Sierra Leone; marutaa@who.int; 12 World Health Organization Regional Office for Africa, Brazzaville P.O. Box 06, Congo; okeibunorj@who.int

**Keywords:** hand hygiene, hand washing, alcohol-based hand rub, compliance, Sierra Leone

## Abstract

Although hand hygiene (HH) is the most effective intervention to reduce the spread of infections, there are limited data on HH facilities, policy, and compliance in sub-Saharan Africa. This cross-sectional study is aimed at assessing HH using the WHO HH self-assessment framework, HH technical reference manual, and a modified infection control self-assessment tool in two hospitals in Sierra Leone. Only 10% and 9% of regional and capital city hospitals had running tap water, respectively. Veronica buckets were the resources for HH in 89% of units in the regional hospital and 92% of units in capital city hospital. Constant supply of soap and alcohol-based hand rub was available in 82% and 68%; and 74% and 79% of units in the capital city and regional hospitals, respectively. Only 10% of the units in both hospitals had hand-drying facilities and functional sinks. Overall HH compliance for the two hospitals was 18.6% and was higher in the regional (20.8%) than the capital city (17.0%) hospitals. The HH levels for the capital city and regional hospitals were 277.5 and 262.5 respectively. Despite the COVID-19 pandemic, there are still challenges with HH compliance in Sierra Leone. It is, therefore, necessary to strengthen the HH multi-modal strategy.

## 1. Introduction

Healthcare-associated infections (HAI) are a major global health problem, causing millions of healthcare-related morbidity and mortality every year [1]. Although there are no comprehensive global data on HAI, their burden in low-income countries is higher than in high-income countries [1]. HAI prolongs hospital stay and increases health care costs [2]. Despites these enormous challenges associated with HAI, there are very few studies investigating hand hygiene in under-resourced health care settings [3,4].

Preventing HAI is thus a crucial intervention that will improve patients’ safety and reduce unnecessary cost and mortality [5,6]. The hands of medical staff are the primary source of the spread of HAI [5,6]. Therefore, proper hand hygiene practices, including washing hands with soap and water or using alcohol-based hand rubs, are the most effective interventions to reduce the spread of infections in healthcare settings [5,7]. Both are simple and quick techniques to prevent HAI, and if implemented correctly, they can save lives, reduce morbidity, and minimize healthcare costs [2]. As part of its global effort to promote the practice of hand hygiene, the World Health Organization (WHO) adapts and adopts the ‘My 5 Moments for hand hygiene’ approach in 2009 [8]. Using this approach, WHO defines the key moments when medical staff should perform hand hygiene: before patient contact, before an aseptic procedure, after bodily fluid exposure risk, after patient contact, and after contact with patient surroundings [8].

Nevertheless, especially in low-income countries, there are still major obstacles hindering routine hand hygiene practices, including the lack of knowledge about hand hygiene among healthcare workers and the unavailability of resources [9,10]. However, the frequent outbreaks of high-risk infectious diseases could have increased the level of knowledge among medical staff of hand hygiene practices [11]. Paradoxically, however, similarly to other essential health services [12,13], the COVID-19 pandemic may have an unprecedented negative impact on the control and prevention of HAI [14].

Since the first confirmed case of COVID-19 in Sierra Leone was reported on 31 March 2020, the government of Sierra Leone has implemented a series of measures including the provision of resources and monitoring compliance to hand hygiene practices in major public hospitals across the country. To understand the impact of these measures, it is necessary to evaluate the hand hygiene facilities and policy and medical staff compliance to hand hygiene practices during the ongoing COVID-19 pandemic in Sierra Leone. This study aimed to assess the facilities and policies available for the practice of hand hygiene and staff compliance in two hospitals in Sierra Leone using the WHO hand hygiene self-assessment framework, hand hygiene technical reference manual, and a modified infection control self-assessment tool

## 2. Materials and Methods

### 2.1. Study Design

The study used a cross-sectional hospital-based design to assess hand hygiene facilities and policy and monitored the hand hygiene compliance of medical staff.

### 2.2. Study Setting

Two hospitals in Sierra Leone were selected for the conduct of the study based on the fact that they are in different geographic regions and likely representative of many tertiary/quaternary hospitals in Sierra Leone and other countries in sub-Saharan Africa where many patients will seek care. Although both hospitals provide quasi-tertiary health care services, they serve large populations in their catchment areas. While one of the hospitals is located in Freetown, the capital of Sierra Leone with a population of one million, the other hospital is located in a regional city about 170 km away from the capital with a catchment population of 606,544 (approximately 8.6% of the Sierra Leonean population) [15]. Both hospitals are owned by the government of Sierra Leone, with similar infrastructure and roughly the same bed capacity. They both provide maternal, medical, surgical, and paediatric services.

In total, 803 medical staffs including 546 (68.1%) nurses, 22 (2.7%) doctors, 20 (2.5%) community health officers, 44 (5.5%) pharmacy personnel, 46 (5.7%) laboratory personnel, 6 (0.7%) radiographers and 119 (14.8%) ancillary staffs are working in the 41 units/wards/departments of the two hospitals.

While the hospital in the capital Freetown has a total of 22 wards, units or departments, the regional hospital has a total of 19 wards, units or departments. Both hospitals have centers for the treatment of high-risk infectious diseases cases. The wards of the two hospitals are designed as open halls that can accommodate 10 to 22 beds. The total number of beds in the regional and the capital Freetown hospitals is 207 and 181 respectively.

A total of 351 medical staff provides services to the regional hospital. Of this, 252 are nurses, 8 are doctors, 8 are pharmacists or pharmacy technicians, 21 are laboratory personnel and 7 are community health officers. The remaining are ancillary staff. The hospital in the capital has a total of 452 staffs including 36 doctors, 294 nurses, 9 pharmacists or pharmacy technicians, 25 laboratory personnel, 13 community health workers and 68 ancillary staff as shown in Table 1. 

Both hospitals have governance structures for infection prevention and control (IPC) practices in line with the prescription of the National Infection Prevention and Control Unit of the Ministry of Health and Sanitation. The overall supervision of IPC services in each hospital is carried out by the ‘IPC focal’. Each unit, ward or department has an ‘IPC linked nurse’ who directly supervises IPC services within their settings. Training and monitoring of hand hygiene compliance are routine in these hospitals.

### 2.3. Hand Hygiene Facilities Assessment

A hospital-based survey on the availability of hand hygiene facilities was conducted in July and August 2021 at the hospitals in the capital city and regional city, respectively using a modified Infection Control Self-Assessment Tool [16]. Two trained nurses from each hospital collected data on the hand hygiene infrastructure (presence of sinks, availability of running water or hand-operated taps), hand hygiene facilities (availability of alcohol-based hand rubs and soap and hand drying facilities) and policies related to hand hygiene services (job aids or posters) through interviews and direct observation in all the units, wards or departments.

### 2.4. Hand Hygiene Compliance

The hand hygiene compliance was assessed through the direct observation of a cross-section of healthcare workers from various disciplines at the patient bedside during routine care. In each hospital, two nurses were trained by the “IPC focal” to conduct hand hygiene compliance assessment in the wards or units including the maternity, medical, children, and surgical wards using the WHO Hand Hygiene Technical Reference Manual (HHTRM) [17]. These well-trained nurses used specialized techniques to prevent inherent limitations and potential biases (such as the Hawthorne effect, where people change their behaviour because they know they are being observed).

In total, 1279 (58.2%) and 919 (41.8%) observations were respectively recorded from the hospital in the Capital City and the regional hospital in September 2021, which surpassed the minimum of 56 observations required for reliable estimates of hand hygiene compliance in 100-bed hospitals as prescribed by the Public health Ontario hand hygiene compliance and observation analysis and standards [18]. All the observations were entered into the standard WHO proforma for hand hygiene compliance assessment [17]. The compliance per facility is determined by the quotients of the observed hand hygiene actions performed when an opportunity occurs and the total number of opportunities. Grading of hand hygiene compliance was arbitrary and it was considered good when greater than 50% per facility [19].

### 2.5. Hand Hygiene Policy

Hand hygiene policy was assessed using the WHO hand hygiene self-assessment framework (HHSAF) to determine the progress on hand hygiene practices, identify gaps and develop actions for improvements [20]. Using a multi-modal approach, the five components of this tool including system change, education and training, evaluation and feedback, reminders in the workplace, and institutional safety climate were assessed by the study physician through interview and direct observation [20].

### 2.6. Data Analysis

Data were analysed using SPSS version 22 and STATA version 16.1. Frequencies and proportions were used to analyse the data on hand hygiene self-assessment framework and hand hygiene facilities.

Hand hygiene compliance was determined as the percentage of observed hand hygiene actions (hand washing and/or use of alcohol-based hand rubs) in the total number of observed opportunities for hand hygiene. Data were presented separately for both hospitals. The chi-square test is used to determine differences in hospital compliance. Univariate and multivariate logistic regression were used to determine any associations between ward, medical staff, and hand hygiene indications.

Parameters with a *p*-value < 0.25 in the univariate logistic regression model were fitted to the multivariable logistic regression model. The results were presented as crude odds ratio after univariate analysis, and as adjusted odds ratio (aOR) following multivariable analysis. A *p*-value < 0.05 at 95% confidence intervals (CI) was considered statistically significant.

### 2.7. Ethical Consideration

We sought permission from the management of the two hospitals to conduct this research and obtained ethical approval from the Sierra Leone Ethical and Scientific Review Committee of the Ministry of Health and Sanitation. All the data collected were stored electronically in a password-protected Epi-collect data platform. No identifier variables were included in the analysis.

## 3. Results

### 3.1. Hand Hygiene Facilities

**Figure 1:**
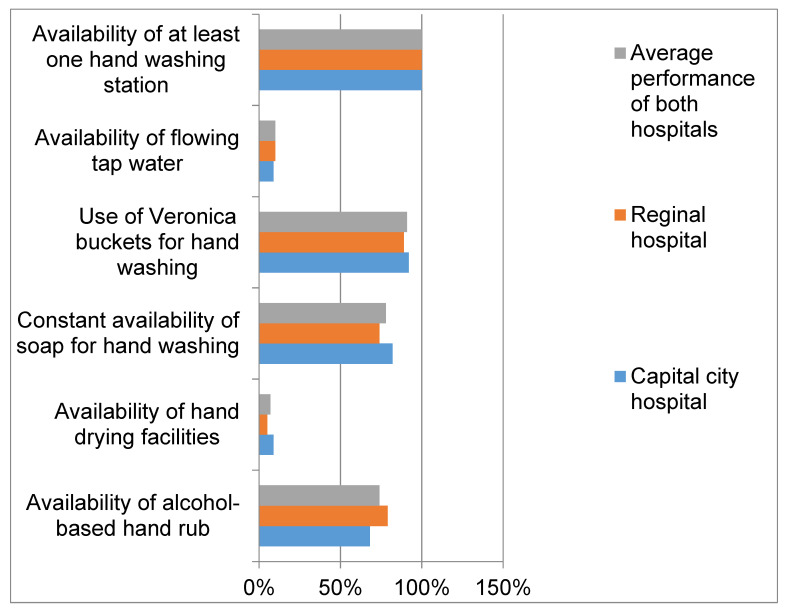
Hand hygiene facilities in the units/wards/departments of two hospitals in Sierra Leone.

All units/wards/departments of the two hospitals had at least one hand-washing station, but only 10% and 9% of the units/wards/departments in the regional and capital cities hospitals had running tap water. Veronica buckets were the resources for hand hygiene in 89% and 92% of the units/wards/departments in the regional and capital city hospitals, respectively. A constant supply of soap was available for hand washing in the 82% of the units in the capital city hospital and 74% of units in the regional hospital. Alcohol-based hand rub was more available in the regional hospital (79%) than the hospital in the capital city (68%). In these two hospitals, less than 10% of the units, wards or departments were equipped with hand-drying facilities. In both hospitals, 90% of all the units/departments/wards were without functional sinks, and the job aids or posters for hand hygiene were not available in 47% of the regional hospital and 41% of the capital city hospital as shown in Figure 1.

There were relatively more hand-washing stations in triage, obstetric wards, and nursing units for high-risk infectious diseases. In contrast to the Intensive Care Unit (ICU) of the hospital in the Capital City which has 5 hand washing stations, the ICU in the regional hospital had only one washing station. Except for the triage and laboratory of the Capital City hospital and the pharmacy and obstetrics units of the regional hospital, the hand washing stations of all other units, wards, or departments used hand-operated (Veronica) buckets. No unit/ward/department uses multiple used towels, and only the maternity unit of the regional hospital and the infectious disease units in either hospital used disposable paper towels for hand drying as shown in Table 2.

### 3.2. Hand Hygiene Compliance

As shown in Table 3, 1279 (58.2%) and 919 (41.8%) observations were respectively recorded from the hospital in the Capital City and the regional hospital. The majority of the hand hygiene observations were made in the surgical ward of the capital city hospital (291, 22.8%) and the maternity ward of the regional hospital (226, 24.6%). In both hospitals, the highest observations were made on nurses (86.1% and 47.2% for capital city hospital and regional hospital, respectively).

The overall hand hygiene compliance rate for the two hospitals was 18.6% and it was higher in the regional hospital (20.8%) than the hospital in the Capital City (17.0%). Compliance was 66% more likely to be observed in the regional hospital than in the capital city hospital [aOR 1.66, 95% CI (1.24–2.23); *p* = 0.001]. Compared to the medical wards in the two hospitals, the surgical wards [aOR 0.49, 95% CI (0.32–0.76); *p* = 0.001], paediatric wards [aOR 0.46, 95% CI (0.30–0.72); *p* = 0.001], and Accident and Emergency (A&E) wards [aOR 0.46, 95% CI (0.29–0.73); *p* = 0.001] were respectively 51%, 54% and 54% less likely to comply with hand hygiene practices. Nurses [aOR 0.63, 95% CI (0.43–0.93); *p* = 0.019] and other health care workers [aOR 0.59, 95% CI (0.37–0.95); *p* = 0.031] of both hospitals were respectively 37% and 41% less likely to observe hand hygiene compared to doctors. Healthcare workers in the two hospitals were 34 times more likely to observe hand hygiene after exposure to body fluids [aOR 34.0, 95% CI (21.5–53.6); *p* < 0.001] and they are about 10 times more likely to observe hand hygiene practices after touching a patient [aOR 9.95, 95% CI (6.8–14.6); *p* < 0.001] than before touching a patient (Table 4).

The highest compliance was registered in ‘other’ wards (a heterogeneous mix of special wards consisting of dental wards and special care baby unit) of the capital city hospital (39.9%) and regional hospital (23.4%). In the capital city hospital, hand hygiene compliance was 64% [aOR 0.36, 95% CI (0.19–0.68); *p* = 0.002] and 70% [aOR 0.30, 95% CI (0.15–0.61); *p* = 0.001] less likely to occur in paediatrics and Accident and Emergency (A& E) wards than in the medical wards, respectively. However, hand hygiene compliance was nearly six times higher in the ‘other’ wards of the capital city hospital than the medical wards [aOR 5.7, 95% CI (3.0–10.9); *p* < 0.001]. There were no statistical differences in hand hygiene practices between wards in the regional hospital as shown in a Table 5.

In the capital city hospital, doctors were the most compliant to hand hygiene practices (30.7%), with nurses being 76% less likely to practice hand hygiene [aOR 0.24, 95% CI (0.14–0.44); *p* < 0.001]. This was not the case in the regional hospital where nurses were the most compliant (23.7%). There was, however, no statistical differences in hand hygiene compliance between medical staff cadre.

The hand hygiene moments where medical staffs in the capital city hospitals are less likely to perform hand hygiene were before touching a patient (0.95%) and before aseptic technique (0.61%). In contrast, hand hygiene compliance was higher after touching patient (38.5%) or patient’s surrounding (7.3%) and after body fluid exposure (45.8%). In addition, the medical staffs were 200 times more likely to perform hand hygiene after body fluid exposure than before touching a patient [aOR 243, 95% CI (68–862); *p* < 0.001].

The regional hospital witnessed similar results. Hand hygiene compliance least occurred before aseptic technique (4.3%) and occurred mostly after exposure to body fluid (87.9%). Hand hygiene compliance was over 60 times higher after body fluid exposure [aOR 66, 95% CI (29–152); *p* < 0.001] and about four times higher after touching patients [aOR 3.9, 95% CI (2.5–6.0); *p* < 0.001] than before touching a patient. The most frequent indication for hand hygiene in both hospitals was before touching a patient; capital city hospital (315, 24.6%) and regional hospital 334 (36.3%). Other details are shown in Table 5. 

### 3.3. Hand Hygiene Levels

**Figure 2:**
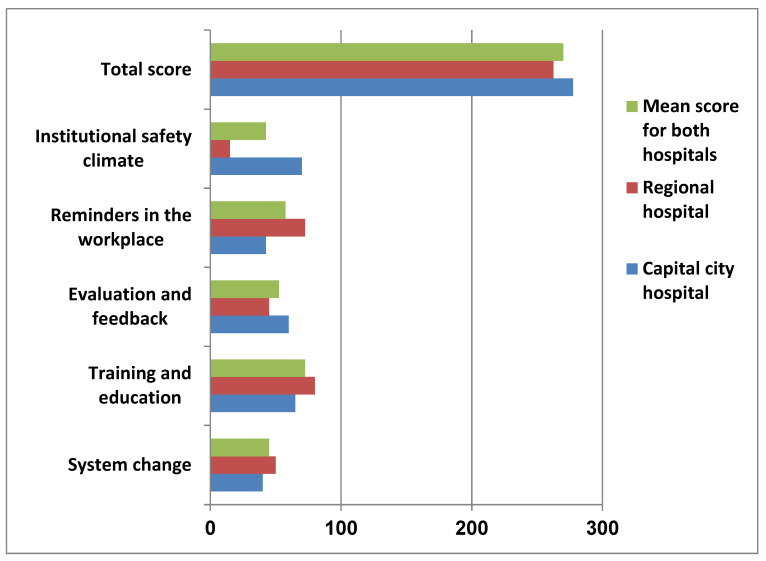
Hand hygiene levels of the two hospitals.

The overall hand hygiene level scores for the Capital City hospital and the Regional hospital were 277.5 and 262.5 respectively. The mean score for both hospitals is 270. Therefore, both hospitals reached the intermediate hand hygiene level. The regional hospital has higher scores in the domains of system change, reminders in the workplace, and training and education. On the other hand, the hospital in the Capital City scored higher in evaluation and feedback and institutional safety climate than the regional hospital as shown in Figure 2. 

## 4. Discussion

In this COVID-19 era, as more resources are used to provide health services, it is expected that hand hygiene practice will be optimal. Owing to resource constraints and infrastructural challenges, however, this does not always apply to all settings. In our study, the use of running tap water was limited to about 10% of all units/wards/department in the two hospitals. The unavailability of running water and the inadequacies in the number of available sinks in many hospitals have compelled many health facilities to use Veronica buckets in hand washing stations. In fact, this innovative method of using Veronica buckets may positively impact hand hygiene practices in under-resourced areas. As shown in this study, approximately 90% of the units/wards/departments of the two hospitals used Veronica buckets in their hand washing stations, because only less than 10% of the sinks are functional. This is not unique to Sierra Leone. In Ghana, Veronica buckets were the main hand washing stations used to prevent the spread of COVID-19 in the main public transportation areas [21]. In this study, unlike reports from Nigeria and Ghana, at least 70% of the wards/units/departments provide a fairly continuous supply of alcohol-based hand rub and soap [19,22]. With the availability of these key resources, it is expected that the hand hygiene compliance rates of medical staff in these two institutions would increase.

However, the overall hand hygiene compliance rate in both hospitals was poor (below the 50% average) and it was even lower in the capital city hospital. Among different medical staff, doctors in the regional hospital have one of the lowest hand hygiene compliance rates, while doctors in the capital city hospital have the highest hand hygiene compliance rates. The well-known phenomenon that doctors are less likely to practice hand hygiene compared with other medical staff [23,24,25] does not apply to the hospital in the capital city. Similarly to a previous study [22], medical staff were more likely to perform hand hygiene with self-protective moments (after touching patients or patient’s surrounding and after body fluid exposure) than patient-protective hand hygiene moments (before touching a patient or before aseptic technique). Thus, reminders and feedback on hand hygiene practice in the workplace should pay special focus to patient-protective hand hygiene moments.

As hand hygiene products were available in many of the units/wards/departments, the lack of support structures such as hand hygiene posters may be a reasonable explanation for the poor performance of hand hygiene in these hospitals or perhaps the lack of knowledge about the importance of hand hygiene may have also played a key role for such low hand hygiene compliance despite the provision of soap and alcohol-based hand rub. Similarly, unsatisfactory behaviour towards hand hygiene practices as highlighted by Głabska et al. may also affect hand hygiene performance in these two hospitals [26]. Therefore, a multimodal approach to hand hygiene is essential to improve hand hygiene practices [23,24]. The hand hygiene self-assessment framework is a WHO tool that determines the level of hand hygiene using a multimodal approach to identify gaps and make recommendations for improvement.

In our study, using this tool, the two hospitals did not meet the advanced hand hygiene standards, so neither had the qualifications for the leadership assessment [20]. The average score of these hospitals was 270 points, which is lower than the global average of 335.1 points in 2011 and 374.4 points in 2015 [27]. However, the lower scores in these hospitals correspond to the average scores of the WHO African Region reported in the global HHSAF assessments of 2011 and 2015 [27. Of interest is that both hospitals scored 50 points or lower in the ‘domain of system change’ (out of 100 points) which ensures that medical institutions have the necessary hand hygiene infrastructure [9, 27].

Our study has a few limitations worthy of mention. One of the limitations of this study is that the WHO HHSAF is a self-reported assessment tool, and the medical staff filling out this form may not be able to provide the true status of the hand hygiene standards in their facilities. Similarly, a comprehensive hand hygiene assessment includes an evaluation of hand hygiene policies, hand hygiene compliance, hand hygiene facilities, and consumption of hand hygiene products. We did not evaluate the consumption of hand hygiene products in this study because there is no accurate estimate of the consumption patterns of hand hygiene products in these hospitals. Although there were efforts to eliminate the Hawthorne effect, some biases might have been introduced during the hand hygiene observation. Nevertheless, our study may be the first to provide more comprehensive evidence for hand hygiene practices in sub-Saharan Africa.

## 5. Conclusions

Despite the on-going COVID-19 pandemic and the availability of soaps and alcohol-based hand rubs in many hospital units, wards or departments, there are still challenges with compliance to the practice of hand hygiene in some hospitals in Sierra Leone. Owing to the limited support structures documented in this study such as hand hygiene posters, it is wise to strengthen the multi-modal approach to hand hygiene practices in these settings.

## Figures and Tables

**Table 1:** Healthcare workers in the provincial and capital city hospitals in Sierra Leone.

Cadre/Bed Capacity/Units	Total		Regional Hospital		Capital City Hospital	
	Freq.	%	Freq.	%	Freq.	%
Bed capacity	388	100.0	207	100.0	181	100.0
Units/wards/department	41	100.0	19	100.0	22	100.0
Number of healthcare workers	803	100	351	100	452	100
Nurses	546	68.1	252	71.8	294	67.9
Doctors	22	2.7	8	2.3	14	3.2
Community health officers	20	2.5	7	2	13	0.3
Pharmacy personnel	44	5.5	8	2.3	36	8.3
Laboratory personnel	46	5.7	21	6	25	5.8
Radiographers	6	0.7	4	1.1	2	0.5
Others	119	14.8	51	14.5	68	15.7

**Table 2:** Hand hygiene facilities in two hospitals in Sierra Leone.

Unit/Ward/Department of the Hospital	Capital City Hospital								Regional Hospital							
	Frq.	Hand Washing Stations	Water Always Available	Hand Operated Tap in the Wash Stations	Use of Liquid Soap	Soap Always Available	Multiple Use Cloth for Hand Drying	Disposable Paper Towel	Frq.	Hand Washing Stations	Water Always Available	Hand Operated Tap Buckets in the Wash Stations	Use of Plain Liquid Soap	Soap Always Available	Multiple Use Cloth for Hand Drying	Disposable Paper Towel
Triage	1	8	Yes	Sink	Yes	Yes	No	No	1	4	Yes	Yes	Yes	Yes	No	No
Laboratories	1	5	Yes	Sink	Yes	Yes	No	Yes	1	1	No	No	No	No	No	No
Accident and Emergency	1	2	Yes	Yes	Yes	Yes	No	No	1	1	Yes	Yes	Yes	Yes	No	No
ICU	1	5	Yes	Yes	No	Yes	No	No	1	1	Yes	Yes	Yes	Yes	No	No
Operating main theatre	1	1	Yes	Yes	Yes	Yes	No	No	1	2	Yes	No	Yes	Yes	No	No
Maternity theatre	NA	NA	NA	NA	NA	NA	NA	NA	1	2	No	No	Yes	Yes	No	Yes
Mortuary	1	2	Yes	Yes	Yes	Yes	No	No	1	1	Yes	No	No	Yes	No	No
Pharmacy	1	1	Yes	Yes	Yes	Yes	No	No	1	1	Yes	Sink	Yes	No	No	No
Physiotherapy unit	1	2	Yes	Yes	Yes	Yes	No	No	NA	NA	NA	NA	NA	NA	NA	NA
SCBU	NA	NA	NA	NA	NA	NA	NA	NA	1	5	Yes	Yes	Yes	Yes	No	No
Male medical ward	2	2	Yes	Yes	Yes	Yes	No	No	1	2	Yes	Yes	Yes	Yes	No	No
Female medical ward	1	2	No	Yes	No	No	No	No	1	1	Yes	Yes	Yes	Yes	No	No
Male surgical ward	2	2	No	Yes	Yes	No	No	No	1	2	Yes	Yes	Yes	Yes	No	No
Female surgical ward	2	2	Yes	Yes	No	Yes	No	No	1	1	Yes	Yes	Yes	Yes	No	No
Dental	1	1	Yes	Yes	Yes	Yes	No	No	0	0	NA	NA	NA	NA	NA	NA
IDPC/MDR-TB ward	1	10	Yes	No	Yes	Yes	No	Yes	1	6	Yes	Yes	Yes	Yes	No	Yes
Ophthalmology	1	1	Yes	Yes	Yes	Yes	No	No	1	2	Yes	Yes	Yes	Yes	No	No
Maternity unit	1	4	Yes	Yes	No	Yes	No	No	1	6	Yes	Sink	Yes	Yes	No	No
Public health	1	1	Yes	Yes	Yes	Yes	No	No	0	0	NA	NA	NA	NA	NA	NA
Research	1	2	Yes	Yes	No	Yes	No	No	0	0	NA	NA	NA	NA	NA	NA
Paediatric ward	1	2	No	Yes	Yes	No	No	No	1	4	Yes	Yes	Yes	No	No	No
Under five	1	2	Yes	No	Yes	Yes	No	No	0	0	No	No	No	No	No	No
Observation ward	NA	NA	NA	NA	NA	NA	NA	NA	1	1	Yes	Yes	Yes	Yes	No	No
X-ray department	NA	NA	NA	NA	NA	NA	NA	NA	1	2	No	Yes	Yes	No	Yes	No
IDCU	1	1	Yes	Yes	Yes	Yes	No	Yes	NA	NA	NA	NA	NA	NA	NA	NA

IDCU: Infectious Disease Control Unit; IDPC: Infectious Disease Prevention and Control; SCBU: Special Care Baby Unit; ICU = Intensive Care Unit; MDR-TB = Multi-drug resistance TB; NA: Not applicable.

**Table 3:** Observed hand hygiene (HH) opportunities in the two hospitals.

Parameter	Total N (%)	Capital City Hospital N (%)	Regional Hospital N (%)
**Total opportunities**	2198 (100%)	1279 (58.2%)	919 (42.8%)
**Ward**			
Medical	353 (16.1)	232 (18.1)	121 (13.2)
Surgical	440 (20.0)	291 (22.8)	149 (16.2)
Paediatric	393 (17.9)	216 (16.9)	177 (19.3)
Maternity	424 (19.3)	198 (15.5)	226 (24.6)
A & E	360 (16.4)	194 (15.2)	166 (18.1)
Others	228 (10.4)	148 (11.6)	80 (8.7)
**Healthcare workers**			
Doctor	250 (11.4)	140 (10.9)	110 (12.0)
Nurse	1535 (69.8)	1101 (86.1)	434 (47.2)
Others	413 (18.8)	38 (3.0)	375 (40.8)
**Indication**			
Before touching a patient	649 (29.5)	315 (24.6)	334 (36.3)
Before aseptic procedure	256 (11.7)	164 (12.8)	92 (10.0)
After exposure to body fluid	221 (10.1)	155 (12.1)	66 (7.2)
After touching a patient	579 (26.3)	304 (23.8)	275 (29.9)
After touching patient surroundings	493 (22.4)	341 (26.7)	152 (15.5)
**Hand hygiene compliance**			
No	1790 (81.4)	1062 (83.0)	728 (79.2)
Yes	408 (18.6)	217 (17.0)	191 (20.8)

**Table 4:** Summary of Hand hygiene compliance in the entire sample (N = 2198).

Parameter	Compliance		Crude Odds Ratio (C.I)	*p*	Adjusted Odds Ratio (C. I)	*p*
	Yes (%) 408 (18.6)	No (%) 1790 (81.4)				
**Hospital**						
Capital city	217 (17.0)	1062 (83.0)	1	-	1	-
Regional	191 (20.8)	728 (79.2)	1.28 (1.03–1.59)	0.023	1.66 (1.24–2.23)	0.001
**Wards**						
Medical	75 (21.3)	278 (78.7)	1	-	1	-
Surgical	63 (14.3)	377 (85.7)	0.62 (0.43–0.89)	0.011	0.49 (0.32–0.76)	0.001
Paediatric	56 (14.3)	337 (85.7)	0.61 (0.42–0.90)	0.013	0.46 (0.30–0.72)	0.001
Maternity	91 (21.5)	333 (78.5)	1.01 (0.72–1.43)	0.942	0.83 (0.55–1.25)	0.382
A & E	45 (12.5)	315 (87.5)	0.53 (0.35–0.79)	0.002	0.46 (0.29–0.73)	0.001
Others	78 (34.2)	150 (65.8)	1.93 (1.33–2.80)	0.001	2.10 (1.35–3.28)	0.001
**Healthcare workers**						
Doctors	63 (25.2)	187 (74.8)	1	-	1	-
Nurses	269 (17.5)	1266 (82.5)	0.63 (0.46–0.86)	0.004	0.63 (0.43–0.93)	0.019
Others	76 (18.4)	337 (81.6)	0.67 (0.46–0.98)	0.038	0.59 (0.37–0.95)	0.031
**Indications**						
HM 1	38 (5.9)	611 (94.1)	1	-	1	
HM 2	5 (1.9)	251 (98.1)	0.32 (0.12–0.82)	0.018	0.39 (0.15–1.01)	0.051
HM 3	129 (58.4)	92 (41.6)	22.5 (14.8–34.4)	<0.001	34.0 (21.5–53.6)	<0.001
HM 4	203 (35.1)	376 (64.9)	8.7 (6.0–12.6)	<0.001	9.95 (6.8–14.6)	<0.001
HM 5	33 (6.7)	460 (93.3)	1.15 (0.71–1.87)	0.561	1.41 (0.86–2.30)	0.175

HM 1 to HM5 = Hand hygiene moments 1 to hand hygiene moments 5.

**Table 5:** Hand hygiene compliance by wards, healthcare workers and indicators in the two hospitals.

Parameters	Capital City Hospital (N = 1279)						Regional Hospital (N = 919)					
	Compliance		OR (C.I)	*p*	aOR (C.I)	*p*	Compliance		OR (C.I)	*p*	aOR (C.I)	*p*
	Yes (%) 217 (17)	No (%) 1062 (83)					Yes (%) 191 (20.8)	No (%) 728 (79.2)				
**Wards**												
Medical	48 (20.7)	184 (79.3)	1	-	1	-	27 (22.3)	94 (77.7)	1	-	-	-
Surgical	34 (11.7)	257 (88.3)	0.51 (0.31–0.82)	0.005	0.58 (0.32–1.05)	0.072	29 (19.5)	120 (80.5)	0.84 (0.47–1.52)	0.566	-	-
Paediatric	21 (9.7)	195 (90.3)	0.41 (0.24–0.72)	0.002	0.36 (0.19–0.68)	**0.002**	35 (19.8)	142 (80.2)	0.86 (0.49–1.51)	0.596	-	-
Maternity	40 (20.2)	158 (79.8)	0.97 (0.61–1.55)	0.901	1.30 (0.71–2.38)	0.393	51 (22.6)	175 (77.4)	1.01 (0.60–1.72)	0.975	-	-
A & E	15 (7.7)	179 (92.3)	0.32 (0.17–0.59)	<0.001	0.30 (0.15–0.61)	**0.001**	30 (18.1)	136 (81.9)	0.77 (0.43–1.38)	0.374	-	-
Others	59 (39.9)	89 (60.1)	2.5 (1.6–4.0)	<0.001	5.7 (3.0–10.9)	**<0.001**	19 (23.8)	61 (76.2)	1.08 (0.56–2.12)	0.813	-	-
**Healthcare workers**												
Doctors	43 (30.7)	97 (69.3)	1	-	1	-	20 (18.2)	90 (81.8)	1	-	1	-
Nurses	166 (15.1)	935 (84.9)	0.40 (0.27–0.59)	<0.001	0.24 (0.14–0.44)	<0.001	103 (23.7)	331 (76.3)	1.4 (0.82–2.39)	0.215	1.3 (0.7–2.4)	0.376
Others	8 (21.1)	30 (78.9)	0.60 (0.25–1.42)	0.246	0.59 (0.20–1.76)	0.345	68 (18.1)	307 (81.9)	0.99 (0.57–1.73)	0.991	0.7 (0.4–1.4)	0.363
**Indications**												
HM 1	3 (0.9)	312 (99.1)	1	-	1	-	35 (10.5)	299 (89.5)	1	-	1	-
HM 2	1 (0.6)	163 (99.4)	0.64 (0.06–6.18)	0.698	1.0 (0.1–9.9)	0.998	4 (4.3)	88 (95.7)	0.39 (0.13–1.12)	0.081	0.4 (0.1–1.1)	0.070
HM 3	71 (45.8)	84 (54.2)	87 (27–286)	<0.001	243 (68–862)	**<0.001**	58 (87.9)	8 (12.1)	61.9 (27.3 –140.3)	<0.001	66 (29–152)	**<0.001**
HM 4	117 (38.5)	187 (61.5)	65 (20–207)	<0.001	125 (36–423)	**<0.001**	86 (31.3)	189 (68.7)	3.9 (2.52–6.0)	<0.001	3.9 (2.5–6.0)	**<0.001**
HM 5	25 (7.3)	316 (92.7)	8.2 (2.5–27.5)	0.001	13 (4–47)	**<0.001**	8 (5.3)	144 (94.7)	0.47 (0.21–1.05)	0.066	0.5 (0.2–1.0)	0.060

N is number of observations for each hospital. HM1-HM5: Hygiene moments 1 to Hand hygiene moments 5.

## Data Availability

The data supporting this article is available in the repository of University of Sierra Leone and will be made available on request to the corresponding author when required.
